# EUS-based shear wave elastography of the spleen for detection of clinically significant portal hypertension

**DOI:** 10.1016/j.igie.2024.09.002

**Published:** 2024-09-07

**Authors:** Jad P. AbiMansour, Jerry Yung-Lun Chin, Eric J. Vargas, Jyotroop Kaur, Barham K. Abu Dayyeh, Ryan J. Law, Vishal Garimella, Michael J. Levy, Andrew C. Storm, Ross Dierkhising, Alina Allen, Vinay Chandrasekhara

**Affiliations:** 1 Division of Gastroenterology and Hepatology, Mayo Clinic, Rochester, Minnesota, USA; 2 Division of Clinical Trials and Biostatistics, Department of Quantitative Health Sciences, Mayo Clinic, Rochester, Minnesota, USA

## Abstract

**Background and Aims:**

A measurement of spleen stiffness has been demonstrated to improve the detection of clinically significant portal hypertension (CSPH). In this study, we evaluated the performance of EUS-guided shear wave elastography (EUS-SWE) for detecting CSPH.

**Methods:**

EUS-SWE measurements of the spleen were compared between patients with and without CSPH. Receiver-operating characteristic curve analysis was performed and quantified by the area under the receiver-operating characteristic curve (AUROC).

**Results:**

Of 142 patients with EUS-SWE spleen measurements, 13 (9.2%) had CSPH and 129 (90.8%) did not. Patients with CSPH had a significantly higher mean spleen stiffness (37.6 ± 8.5 kPA vs 29.1 ± 9.9 kPA, *P* = .003). The AUROC was .74.

**Conclusions:**

SWE is a promising technology that can readily be incorporated into standard EUS examination for assessment of portal hypertension.

Portal hypertension is defined by an increased venous pressure gradient across the liver, which most commonly develops in the setting of cirrhosis.^1^ A normal portal pressure gradient (PPG) across the liver is <5 mm Hg. When the PPG reaches 10 mm Hg, the risk of adverse events increases significantly and is referred to as clinically significant portal hypertension (CSPH).^2^ Sequelae of CSPH can include varices, ascites, and cardiopulmonary dysfunction. These adverse events represent the primary cause of death in patients with cirrhosis and are common indications for transplantation. Therefore, the detection of CSPH is of critical importance for informing the management and monitoring of patients with advanced liver disease.

The criterion standard for detection of CSPH is direct PPG measurement, which can be obtained through percutaneous or endoscopic approaches.^3^ A PPG measurement can also be obtained indirectly, but all techniques require vascular access and are relatively invasive. There has been interest in evaluating for CSPH using noninvasive tests. Several methods for evaluating liver stiffness have emerged, including transient elastography (TE), transabdominal US shear wave elastography (SWE), and magnetic resonance elastography (MRE). One study of liver stiffness measured by TE reported a sensitivity of 87% and specificity of 85% for the detection of CSPH.^4^ However, limitations of this approach include confounders that can contribute to elevated measurements (eg, inflammation) and reliability issues related to examination operator and body mass index.

Spleen stiffness measurement is another potential method to evaluate for CSPH, with a sensitivity of 98% and specificity of 74% in 1 study that used TE.^5^ Recent upgrades of EUS processors now permit the use of SWE to provide quantitative assessment of liver stiffness^6^ but also the ability to evaluate spleen stiffness. Therefore, the primary aims of this study were to evaluate the performance of spleen stiffness measured by EUS-guided SWE (EUS-SWE) for the detection of CSPH and define test characteristics for future investigation.

## Methods

Adults who were undergoing a clinically indicated EUS were prospectively enrolled to undergo EUS-SWE during their endoscopy at a single tertiary care center between August 2020 and March 2023 with referrals generated from a variety of gastroenterology subspecialties and general practitioners. A cohort of patients who underwent EUS-SWE of the spleen were identified and included for analysis. The study was deemed exempt by the Mayo Clinic Institution Review Board (IRB no. 20-006775). Sedation was performed at the discretion of the clinical anesthesia team per standard of care. During EUS (GF-UCT180; Olympus, Tokyo, Japan), the spleen was visualized transgastrically with a linear-array echoendoscope without exerting excessive pressure on the splenic capsule. SWE mode was activated on the US processor (ARIETTA 850; Fujifilm, Tokyo, Japan) that was equipped with optional SWE software (Fujifilm). The optimal region of interest was set at 1 to 1.5 cm from the probe while avoiding large ductal or vascular structures.^6^

SWE uses an acoustic radiation force to generate shear waves in the spleen within a region of interest. Tissue displacement is measured, and the local shear wave speed is calculated. The shear wave velocity is used to generate an elastic modulus measured in kilopascals (kPa) that estimates stiffness. The spleen parenchyma was visualized without exerting excessive pressure on the spleen capsule. SWE mode was then activated, and the optimal region of interest was set to between 1 and 1.5 cm from the EUS probe while avoiding large ductal or vascular structures. Consecutive readings were generated with careful attention taken to ensure measurements were obtained during the stationary phase of patient respiration to minimize motion artifact. Individual readings with a reliability index >60% were recorded, whereas those <60% were deleted and remeasured until 10 reliable readings were obtained.^7^ This technique was also used to generate 10 reliable readings from the left and right lobes of the liver, and mean liver stiffness was obtained based on validation data from benchtop models and preclinical studies.^6^ If patients had undergone MRE within 6 months of their endoscopic procedure, liver stiffness obtained by this modality was also recorded. Technical considerations for obtaining liver stiffness by MRE have been well described previously.^8^

The presence of portal hypertension was defined by clinical evidence of portal hypertension (eg, hepatic encephalopathy, spontaneous bacterial peritonitis, presence of esophageal varices) or by imaging evidence of portal hypertension (eg, portosystemic shunting, varices). Patients with intrinsic liver disease were ultimately diagnosed as either having or not having CSPH by a clinically treating hepatologist. EUS-SWE measurements of the spleen were compared between patients with portal hypertension versus those without portal hypertension using a Kruskal-Wallis rank sum test with significance defined as *P* ≤ .05. A receiver-operating characteristic (ROC) curve analysis was performed to quantify diagnostic accuracy for CSPH and quantified by the area under the ROC curve (AUROC). Cutoff values were assessed using Youden’s index. Univariate linear regression was performed to evaluate whether spleen stiffness was correlated to liver stiffness when measured with the same EUS-SWE technique.

## Results

Of 142 patients who underwent EUS-SWE of the spleen, 13 (9.2%) had CSPH and 129 (90.8%) did not. There were no significant differences in patient age, sex, or body mass index (Table 1). Eighty-four patients (59.2%) were sedated with general anesthesia and 58 (40.8%) with monitored anesthesia care. Most patients with portal hypertension had liver disease attributed to either alcohol or metabolic dysfunction (8/13, 61.6%). One patient (7.1%) had noncirrhotic portal hypertension.

**Table 1:** Clinical and procedural characteristics of patients with or without clinically significant portal hypertension

	No clinically significant portal hypertension (n = 129)	Clinically significant portal hypertension (n = 13)	*P* value
Female sex	73 (56.6)	8 (61.5)	.73
Mean age, y (SD)	59.3 (14.3)	62.2 (15.4)	.48
Mean body mass index, kg/m^2^ (SD)	28.2 (6.0)	29.2 (5.0)	.55
Reported history of liver disease			<.001
None	105 (81.4)	1 (7.7)	
Alcohol	3 (2.3)	3 (23.1)	
Metabolic-associated steatotic liver disease	9 (7.0)	5 (38.5)	
Primary sclerosing cholangitis	9 (7.0)	2 (15.4)	
Other (eg, viral, primary biliary cirrhosis, autoimmune hepatitis)	3 (2.3)	2 (15.4)	
Mean Fibrosis-4 index score (SD)	1.7 (1.8)	4.7 (3.0)	<.001
Mean EUS liver stiffness, kPA (SD)	15.9 (9.9)	27.0 (13.8)	<.001
EUS spleen stiffness			
Mean elastic modulus, kPa (SD)	29.1 (9.9)	37.6 (8.5)	.003
Mean shear wave velocity, m/s (SD)	3.0 (.63)	3.3 (.8)	.36
Mean reliability index, % (SD)	85.1 (12.3)	74.0 (23.9)	.12

Values are n (%) unless otherwise defined.

*kPa,* Kilopascals; *SD*, standard deviation.

**Figure 1:**
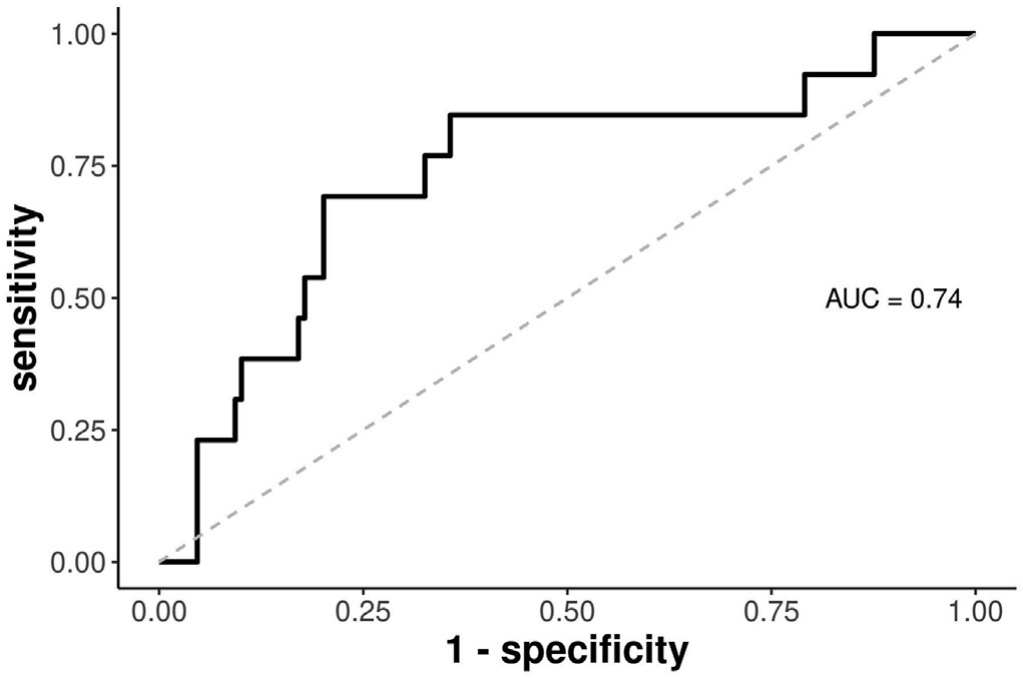
Receiver-operating characteristic curve for the detection of clinically significant portal hypertension. *AUC*, Area under the curve.

**Figure 2:**
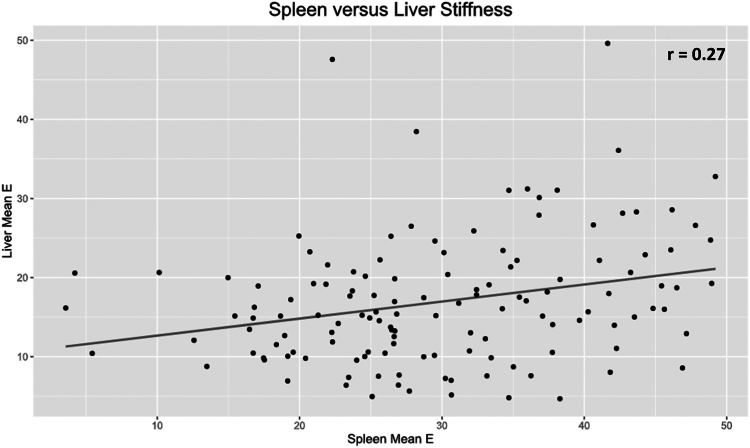
Correlation between mean spleen and liver stiffness measurements obtained using endosonographic shear wave elastography. *E*, Elastic modulus.

Patients with CSPH had a significantly higher mean spleen stiffness when compared with patients without CSPH (37.6 ± 8.5 kPA vs 29.1 ± 9.9 kPA, *P* = .003). There was no significant difference in shear wave velocity or reliability index between the 2 cohorts. The median number of measurements per patient was 12 (interquartile range, 11-17). The AUROC was .74 (Fig. 1). The test cutoff that maximized performance characteristics was 37.6 kPa with a 69.2% sensitivity and 79.8% specificity. Univariate linear regression found that spleen stiffness was significantly correlated with liver stiffness measured obtained using EUS-SWE (β = .21 kPa, *P* = .001) and MRE (β = .08 kPa, *P* = .003). The correlation between EUS-SWE spleen and liver stiffness is shown in Figure 2.

## Discussion

Determining whether a patient has CSPH is critical for the management of chronic liver disease. Advancements in the noninvasive quantification of liver fibrosis, whether with transabdominal US, MRI, or EUS, have allowed for robust risk stratification. However, these measurements are ultimately a surrogate marker for increased portovenous pressure and its downstream sequelae. Interestingly, a meta-analysis of 16 studies consisting of 1892 patients suggested that spleen stiffness performed better than liver stiffness for the prediction of esophageal varices.^9^ Another recent meta-analysis that included 1245 patients from 14 studies showed that spleen stiffness measured with TE or SWE had a 90% to 98% positive predictive value for the presence of CSPH.^10^ Yet, most studies in these cohorts used transabdominal TE and SWE with no data on EUS-derived measurements.

The data from this current exploratory study show that obtaining EUS-SWE measurements from the spleen is feasible, reliable, and correlates with the presence of CSPH. The operating characteristics were favorable with an AUROC of .74. This result is similar to previously published studies using other EUS-based technology, including splenic measurements with strain ratios and strain histograms that found an AUROC between 81% and 84% for the detection of cirrhosis and portal hypertension.^11^ Sensitivity and specificity were modest in the patient population reported in this study, with a specificity of 80% at a cutoff of 37.6 kPa. This cutoff can easily be adjusted to improve the specificity and should be corroborated on future investigation with larger cohorts.

Spleen stiffness appears to correlate with liver stiffness regardless of the modality in which liver stiffness was obtained and may provide similar ability to risk stratify patients, particularly those with sinusoidal disease, which represented most patients in the CSPH cohort. There are certain clinical scenarios in which spleen stiffness may outperform liver stiffness in the detection of CSPH and related adverse events, particularly in those without intrinsic liver disease and prehepatic CSPH etiologies.^9^ Further studies are needed to determine whether this holds true for EUS-SWE–derived measurements. Evaluation of spleen stiffness may also help overcome the heterogenous nature of many intrinsic liver diseases that can impact liver parenchyma heterogeneously, a phenomenon that becomes more pronounced as liver disease progresses.^12^

As with liver stiffness, many diagnostic modalities have the ability to quantify splenic stiffness, and there is a paucity of comparative data given the relative novelty of EUS-SWE. Test characteristics elucidated by this cohort can help inform comparative studies that use transabdominal stiffness measurements. Data from this cohort suggest EUS-derived measurements are fairly reliable with a median of 12 measurements taken to obtain 10 reliable readings. Similar information on transabdominal SWE is sparse, and variable protocols make comparing the number of readings and time required to obtain reliable data challenging.^13^^,^^14^

There are some theoretical advantages to obtaining these measurements transgastrically during an EUS examination. For example, it can be readily performed in individuals with significant subcutaneous and intra-abdominal fat, especially those with central obesity and concern for metabolic dysfunction. This was not readily assessed in this cohort given the similar body mass indices across both groups. In our experience, the spleen is also less impacted by cardiac motion or respiratory variation than the liver, which may allow for more reliable readings. Additionally, EUS is often used for presurgical planning, thus making SWE an attractive, easy option if it is able to detect occult CSPH and aid in clinical decision-making. Endoscopy is also routinely performed for the surveillance and management of patients with adverse events from liver disease (eg, esophageal and gastric varices). Information on the degree of portal hypertension may influence further management decisions.

Limitations of this study include data collection from patients undergoing EUS examination for different indications, lack of blinding of the endoscopists, and small number of patients with CSPH, which limits generalizability. It should also be noted that the criterion standard for portal pressure measurement is direct measurement of the PPG, which was not used in this study in favor of a clinical and radiologic definition that is more consistent with routine clinical practice. However, all patients with CSPH were assigned the diagnosis by a clinical hepatology team that was independent of the study team.

EUS-SWE measurements of the spleen have the potential to provide key information on the status of a patient’s liver disease during endoscopy and can contribute to the field of endohepatology where in 1 session variceal screening and multiple EUS-guided hepatic interventions can be performed. The technique adds minimal risk and does not require a skillset beyond diagnostic EUS. Additional prospective studies are needed to validate and optimize the diagnostic performance while also determining how spleen stiffness correlates to decompensating events like variceal bleeding. The optimal use of EUS-SWE technology would detect CSPH in patients before exhibiting clinical or imaging findings or in those with conflicting results. Ultimately, the goal is to use the diverse tools available to gastroenterologists to not only evaluate the status of a patient’s liver disease but also provide prognostic information on the risk of progression and decompensation, with an aim to provide more individualized and appropriate care.

## Disclosure

The following authors disclosed financial relationships: B. K. Abu Dayyeh: Research support from Apollo, Aspire, Cairn Diagnostics, and Spatz; consultant for BFKW, Boston Scientific, DyaMx, Endogastric Solutions, Metamodix, and USGI; speaker for Endogastric Solutions, Johnson & Johnson, Medtronic, and Olympus; teacher for Olympus. R. Law: Consultant for Medtronic and ConMed. A. C. Storm: Consultant for Apollo Endosurgery, Endo-TAGSS, Enterasense, Erbe, GI Dynamics, and Olympus; research support from Apollo Endosurgery and Boston Scientific. A. Allen: Research support to Mayo Clinic from Novo Nordisk, Pfizer, and Target Pharma; consultant for Novo Nordisk and Pfizer. V. Chandrasekhara: consultant for Boston Scientific and Covidien; research support from Micro-Tech Endoscopy and STARmed; stock shareholder in Nevakar Corporation. All other authors disclosed no financial relationships.
